# Humic acid inhibits HBV-induced autophagosome formation and induces apoptosis in HBV-transfected Hep G2 cells

**DOI:** 10.1038/srep34496

**Published:** 2016-10-06

**Authors:** Kishor Pant, Ajay K. Yadav, Parul Gupta, Abhishek Singh Rathore, Baibaswata Nayak, Senthil K. Venugopal

**Affiliations:** 1 Faculty of Life Sciences and Biotechnology, South Asian University, Akbar Bhawan Chanakyapuri New Delhi, India; 2 Department of Gastroenterology and Human Nutrition, All India Institute of Medical Sciences, New Delhi, India

## Abstract

Hepatitis B Virus (HBV) utilizes several mechanisms to survive in the host cells and one of the main pathways being autophagosome formation. Humic acid (HA), one of the major components of Mineral pitch, is an Ayurvedic medicinal food, commonly used by the people of the Himalayan regions of Nepal and India for various body ailments. We hypothesized that HA could induce cell death and inhibit HBV-induced autophagy in hepatic cells. Incubation of Hep G2.2.1.5 cells (HepG2 cells stably expressing HBV) with HA (100 μM) inhibited both cell proliferation and autophagosome formation significantly, while apoptosis induction was enhanced. Western blot results showed that HA incubation resulted in decreased levels of beclin-1, SIRT-1 and c-myc, while caspase-3 and β-catenin expression were up-regulated. Western blot results showed that HA significantly inhibited the expression of HBx (3-fold with 50 μM and 5-fold with 100 μM) compared to control cells. When HA was incubated with HBx-transfected Hep G2 cells, HBx-induced autophagosome formation and beclin-1 levels were decreased. These data showed that HA induced apoptosis and inhibited HBV-induced autophagosome formation and proliferation in hepatoma cells.

Hepatitis B Virus (HBV) infection, one of the main causes of liver cancer, is responsible for chronic infections in approximately 350 million people worldwide, (~200 million in China and 1.25 million in the U.S.). HBV infection may persist for the complete life of the host, leading to many dreadful consequences in liver (e.g. fibrosis, cirrhosis and hepatocellular carcinoma (HCC))^1^,^2^. There is no available treatment so far, which can completely eradicate the viral infection in chronic HBV patients. Adefovir, lamivudine, telbivudine, and entecavir are the approved chemotherapeutic drugs, which inhibit viral replication by targeting the DNA polymerase of HBV. However, the long-term treatment using these drugs may develop a drug-resistant virus which could be a problematic condition for patients^3^.

HBV modulates several intracellular mechanisms, such as autophagy, to survive in the host. Autophagy is a salvage catabolic process in which cells eliminates proteins and damaged organelles to maintain the cellular homoeostasis. HBV has been reported to enhance the autophagy formation for its support in replication in hepatoma cells without promoting the degradation of lysosomes^4^,^5^,^6^. Small surface proteins (HBsAg) and hepatitis B virus-X protein (HBX) are required to induce autophagy in the infected cells^6^,^7^. Disturbance of the autophagy and induction of the cell death in HBV-infected cells might inhibit the virus replication and may be an added therapeutic approach against this virus^7^.

Humic Acid (HA) is a naturally occurring organic material contains acidic, carboxy and phenol -OH functional groups and it is a product of the decomposition of plant and/or animal residues by microorganisms^8^,^9^. HA has been shown to possess a number of medicinal applications such as anticancer and anti-inflammatory properties^9^. Moreover, humic compounds have been reported to be effective against Herpes Simplex Virus-1 (HSV-1)^10^, Human Immune deficiency Virus (HIV)^11^,^12^ and Cytomegalovirus (CMV)^11^.

Mineral Pitch (MP; Shilajit) is a humic matter age-old Ayurvedic medicine collected mainly from the Himalayan regions of India, Bhutan, Pakistan and Nepal. The humic matter is one of the major constituents of MP, which has been reported as an anti-inflammatory and antioxidant drug, but specific assays are needed to vouch for MP as a wide spectrum remedy^12^. Recently MP, collected from the Nepal, has been reported to be effective against HSV, human cytomegalovirus, human respiratory syncytial virus, human rotavirus, and vesicular stomatitis virus^11^,^12^.

The investigation for new compounds with antiviral properties is still needed, since there is a huge demand of cost-effective and suitable drugs for the HBV management and clearance. In this study, we show for the first time that HA to possess anti-HBV properties, by inhibiting HBV-induced autophagosome formation and inducing apoptosis in the HBV expressing cells.

## Results

### HA induces apoptosis and inhibits proliferation in Hep G2.2.1.5 cells

**Figure 1:**
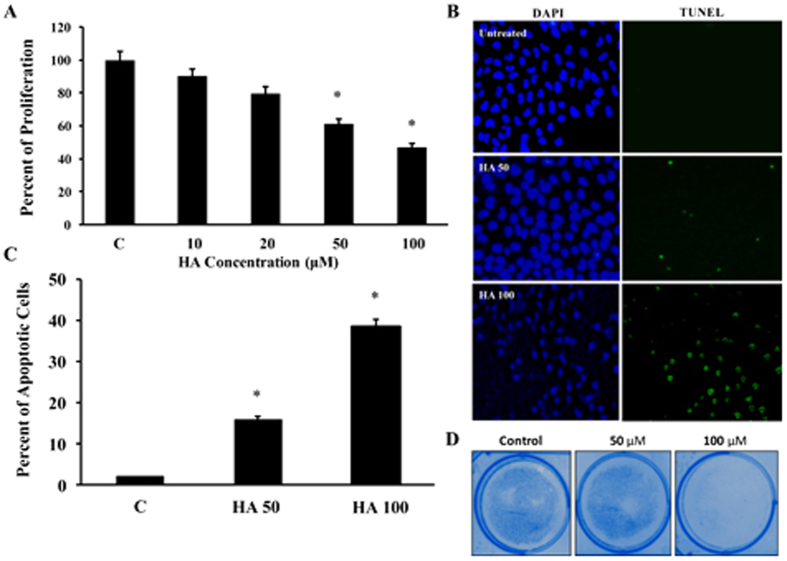
(**A**) Hep G2.2.1.5 cells were cultured with different concentrations of HA (10, 20, 50 and 100 μM) for 24 hours and the cell proliferation assay was performed by MTT assay to check the cell viability (n = 3; *p < 0.05). (**B**) Apoptosis induction by HA was measured in Hep G2.2.1.5 cells by incubating with 50 and 100 μM of HA using TUNEL assay and the representative picture from 3 experiments is shown. (**C**) The total number of positive cells were counted in at least 10 different high power fields and the average is presented. (n = 3; *p < 0.05). (**D**) The colony formation assay was performed in 60 mm dishes with 50 or 100 μM HA (n = 3).

Previous studies have shown that HA possesses anti-cancer activities^13^,^14^. First, the effect of HA on cell proliferation was determined by MTT assay as described in Materials and methods. The results showed that the inhibitory concentration 50 (IC50) concentration of HA was 100 μM and the proliferation was significantly inhibited to 38% and 48% with 50 and 100 μM concentrations of HA respectively (Fig. 1A). Very low concentrations of HA (10 and 20 μM) did not have any significant effects. Hence, for all the experiments both 50 and 100 μM concentrations were used. Incubation of HA with Hep G2.2.1.5 cells increased the induction of apoptosis in a dose dependent manner (15.8% and 38.3% in HA 50 and 100 μM respectively, Fig. 1B,C). Colony formation assay was also performed and was found that the number of colonies of the cells were decreased with increasing concentrations of HA (Fig. 1D).

### HA induces cell death via the caspase-3 activation

**Figure 2:**
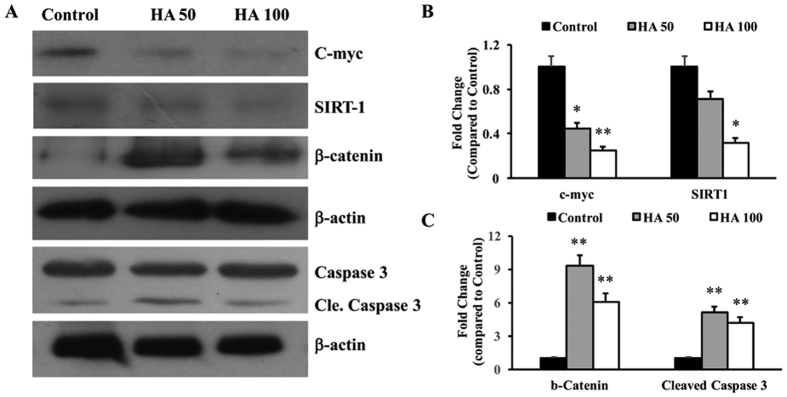
(**A**) Total cellular protein was isolated from the HA (50 and 100 μM) treated Hep G2.2.1.5 cells and Western blots were performed for c-myc, SIRT-1, β-catenin and cleaved caspase 3. In all experiments β-actin was used as an internal control. The picture shown is a representative of 3 experiments. Cle. Caspase – Cleaved caspase 3. (**B,C**) The band intensities were quantified using Image J software and the data are presented. (n = 3; *p < 0.05, **p < 0.01).

Hep G2.2.1.5 cells were cultured with different concentrations of HA (50 and 100 μM) for 24 hours. The total cellular protein was isolated, estimated and 40 μg/well was loaded onto the SDS-PAGE gels and the expression of c-myc, SIRT-1, β-catenin and β-actin were determined. The results showed that both c-myc and SIRT-1 were significantly inhibited, while β-catenin levels were up-regulated (Fig. 2A). The cleaved caspase-3 was also determined using Western blots and found that the levels were increased with HA treatment (Fig. 2A). The band intensities were determined using Image J software and the data are presented in Fig. 2B,C. These results indicate that HA induces apoptosis and inhibits cell proliferation.

### HA inhibits the autophagosome formation in HBV infected cells

**Figure 3:**
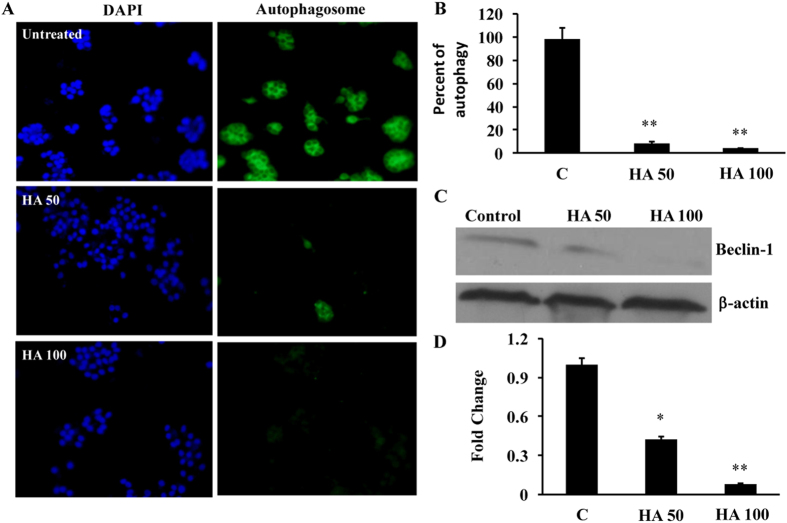
(**A**) Autophagosome formation was measured by culturing cells with or without HA (50 and 100 μM) for 24 hours. The picture is a representative of 3 experiments. (**B**) The positive cells were counted in at least 10 different high power fields and averaged and plotted. (n = 3; **p < 0.01). (**C**) Western blot for beclin-1 and β-actin were performed in Hep G2.2.1.5 cells. (**D**) The image intensities of beclin-1 and β-actin expression were quantified using Image J software and the average was presented. The untreated control cells were taken as 1. (n = 3; *p < 0.05, **p < 0.01).

Recently we have shown that MP induces apoptosis via up-regulation of miRNA-22 in Huh 7 cells^14^. Previously, we had also shown that HBx induces autophagosome formation in hepatic cells^15^. Hence to check whether HA inhibits autophagosome formation in hepatic cells, Hep G2.2.1.5 cells (HBV stably expressing cells) were cultured in 6 well plates and autophagosome formation assay was performed. The results clearly indicated that addition of HA inhibited autophagosome formation in these cells (Fig. 3A). The cells were counted in at least 10 different high-power fields, averaged and quantified and found that there was a significant reduction in the number of autophagosome formation in these cells (Fig. 3B). Beclin-1 is one of the key proteins involved in inducing apoptosis. Hence, the expression of beclin-1 was determined and found that there was a significant inhibition of beclin-1 in HA-treated cells (50 and 100 μM) and the densitometry was performed using Image J software and there was a 48% and 83% inhibition of beclin-1 expression was found (Fig. 3D).

### HA inhibits HBx protein expression in Hep G2.2.1.5 cells

**Figure 4:**
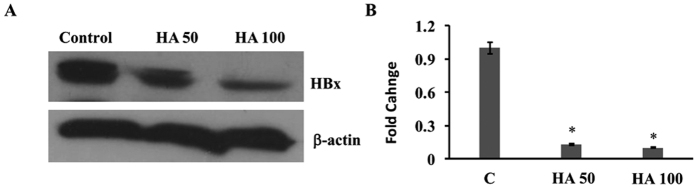
(**A**) Hep G2.2.1.5 cells were cultured with or without HA (50 and 100 μM) and Western blots for HBx and β-actin were performed. (**B**) The band intensities were quantified and presented from 3 different experiments (n = 3; *p < 0.01).

Our previous study had shown that HBx induced the expression of beclin-1 in hepatic cells and inhibition of HBx resulted in inhibition of autophagy in these cells^15^. Hence, we wanted to test whether HA inhibits autophagy via inhibition of HBx in hepatic cells. The protein expression data showed that the incubation of HA (50 and 100 μM) with Hep G2.2.1.5 cells resulted in a significant inhibition of HBx protein expression (Fig. 4A). The data from three different experiments were quantified using Image J software and are presented in Fig. 4B.

### HA inhibits HBV DNA and HBsAg in Hep G2.2.1.5 cells

**Figure 5:**
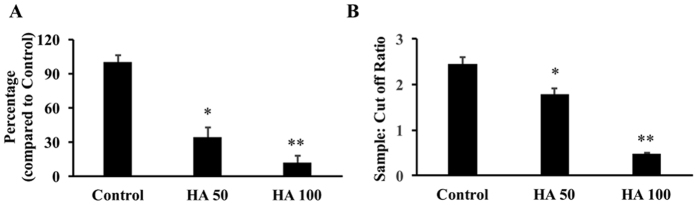
Hep G2.2.1.5 cells were incubated with 50 and 100 μM concentrations of HA for 24 hours. (**A**) The HBV DNA was determined using real time PCR reaction (n = 3; *p < 0.05, **p < 0.01). (**B**) HBsAg levels were measured using ELISA kit as described in Materials and methods (n = 3; *p < 0.05, **p < 0.01). Samples with ratio values < 1 were considered negative and with ratio values >1 were considered positive.

The ability of HA to inhibit HBV DNA and HBsAg in Hep G2.2.1.5 cells was determined by real time PCR and ELISA respectively. The data showed that there was a significant inhibition of HBV DNA to 34.6% and 12% compared to control Hep G2.2.1.5 cells in 50 and 100 μM HA treated cells respectively (Fig. 5A). Qualitative ELISA was done for the presence of HBsAg in the cell culture supernatants. Incubation of cells with 50 and 100 μM HA resulted in a significant inhibition of HBsAg levels (Fig. 5B). Samples with ratio values <1 were considered negative and with ratio values >1 were considered positive. The percentage inhibition was determined on the basis of sample: cut off value. There was a 23% and 80.83% inhibition of HBsAg was observed with the incubation of 50 and 100 μM HA with Hep G2.2.1.5 cells respectively.

### HA inhibits HBx-induced autophagosome formation in Hep G2 cells

**Figure 6:**
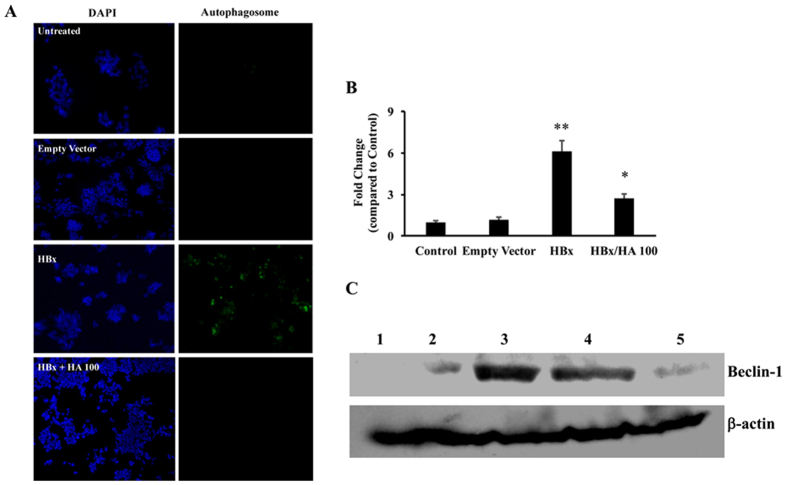
(**A**) Hep G2 cells were transfected with HBx followed by incubation with HA (100 μM) for 24 hours. Autophagosome formation was measured as described in Materials and methods. (**B**) At least 10 different high power fields were counted, the positive cells were averaged and the fold change over control was calculated (n = 3; *p < 0.05 compared to HBx, **p < 0.01 compared to control). (**C**) Western blots were performed in the beclin-1 protein isolated from the HBx transfected HA (50 or 100 μM) incubated Hep G2 cells. Lane 1, control; lane 2, empty vector transfected cells; lane 3, HBx transfected cells; lane 4, HBx transfected and HA (50 μM) incubated cells; and lane 5, HBx transfected and HA (100 μM) incubated cells. Empty vector was used as transfection control in all the experiments. β-actin was used as an internal control (n = 3).

Next, to confirm these findings, Hep G2 cells were transfected with HBx containing plasmid and then they were incubated with either 50 or 100 μM concentration of HA. After 24 hours, the cells were isolated either for autophagosome formation assay or Western blots for signalling molecules. In all these experiments, the 50 μM HA did not result in any significant changes. The results showed that HBx induced the autophagosome formation, while the addition of 100 μM HA inhibited the autophagosome formation in these cells (Fig. 6A). These results were quantified in at least 10 different high-power fields and the mean values are presented in Fig. 6B. Next, the expression of beclin-1 was determined in HBx over-expressing cells with or without the addition of HA. There was a significant increase in beclin-1 in HBx transfected cells, which was inhibited in HA-treated cells (Fig. 6C).

**Figure 7:**
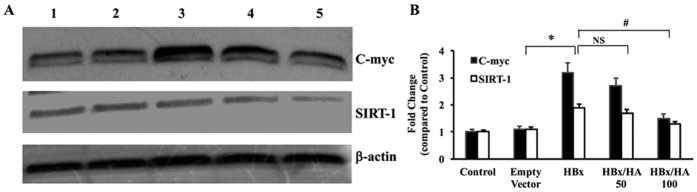
(**A**) Hep G2 cells were transfected with HBx and were incubated with HA (50 or 100 μM). Western blots were performed for C-myc and SIRT-1 from total cellular protein. Lane 1, control; lane 2, empty vector transfected cells; lane 3, HBx transfected cells; lane 4, HBx transfected and HA (50 μM) incubated cells; and lane 5, HBx transfected and HA (100 μM) incubated cells. Empty vector was used as transfection control in all the experiments. β-actin was used as an internal control (n = 3). (**B**) The band intensities were quantified using Image J software and the average from 3 experiments are plotted as fold change (*p < 0.05 compared to control; ^#^p < 0.05 compared to HBx transfected cells).

Next, the expression of c-myc and SIRT-1 was determined in Hep G2 cells expressing HBx protein with or without HA. There was a significant increase in the expression of c-myc and SIRT 1 proteins in HBx expressing cells, while incubation of these cells along with 100 μM HA inhibited the expression (Fig. 7A). In all these Western blot experiments β-actin was used as an internal control. These results were confirmed by quantitating the band intensities using Image J software (Fig. 7B).

## Discussion

Humic acid is one of the active ingredients of MP, a dark brown coloured humic matter that drips out of the high altitude rocks (above 1000 meters) during summer months. Recently we have shown that the MP possesses anti-proliferative and pro-apoptotic properties in Huh7 cells^14^. Previous studies have shown that humic acid can induce apoptosis in cancer cells^16^. A synthetic humate analogue was shown to inhibit HIV-1 in cell culture system^10^. But there is no data available on anti-HBV properties of HA. In this report for the first time, we showed that HA significantly inhibited HBx protein expression, HBV-induced proliferation, and autophagosome formation while induced apoptosis in hepatic cancer cells.

The cell proliferation assay showed that there was a significant inhibition of cell viability when incubated with 50 μM and above concentrations HA. Preliminary experiments were carried out with HA concentrations of HA and IC50 was found to be 100 μM. The lower concentrations of HA (10 and 20 μM) did not have any effects on proliferation. Hence, all the experiments were planned with two concentrations of HA (50 and 100 μM). Incubation of Hep G2.2.1.5 cells with HA resulted in a significant induction of apoptosis. This data is in agreement with the previous reports that HA induced apoptosis in HL-60^16^ and HIT-T15 cells^17^. We have recently shown that MP induces apoptosis in Huh7 cells^14^. HA could be one of the major constituents of that compound, which could induce the apoptosis. Most of the anti-cancer compounds also possess the anti-proliferative activities. Next, the effect of HA on cell proliferation was tested. There was a significant inhibition of proliferation in HA incubated cells. The expression of proto-oncogene c-myc and SIRT-1 were significantly inhibited by HA. Although there was some inhibition of SIRT-1 with 50 μM of HA, but was not significant. SIRT-1 was shown to induce proliferation in human melanoma cells^18^ and C2C12 myoblast cells^19^. Our data also showed that there was an upregulation of β-catenin in HA incubated cells. Previous data show that inhibition of β-catenin resulted in increased proliferation of colon cancer cells^20^. Hence, incubation of HA not only induces apoptosis and it also inhibits proliferation via inhibiting c-myc and SIRT-1, while upregulating β-catenin.

HBV is one of the major threats to the human society, which is shown to be one of the key regulators of the liver cancer^21^,^22^. Viral protein, HBx, can induce proliferation by inducing c-myc and autophagy by enhancing the expression of the beclin-1, thus helping the virus for its pathogenesis and survival^23^. Previously we have shown that HBV can induce proliferation via HBx-mediated miRNA-21 upregulation^24^,^25^. Recently we have also shown that HBx induces autophagosome formation via inhibition of miRNA-30a in hepatic cells^15^. There are few reports that show humic matters and its analogue could inhibit many viruses (including, HIV and HSV) in cell culture model system^10^,^11^. Hence, it was hypothesized that HA could inhibit HBV-induced autophagy and might induce apoptosis in hepatic cancer cells. To test this hypothesis, Hep G2.2.1.5 cells were incubated with HA and the effect on HBx protein and autophagy were determined. HepG2 2.1.5 cells are widely used for the evaluation of anti-HBV drugs, and they contain multiple copies of the HBV genome. Incubation of cells with HA resulted in a significant inhibition of HBx protein expression. As demonstrated, HA effect on HBx was dose-dependent. Our results indicated that HA also inhibited both HBV DNA and HBsAg levels in Hep G2.2.1.5 cells. These data confirm the findings that HA possesses anti-viral activities. To confirm the findings that HBx-induced autophagosome was inhibited by HA, first HBx was over-expressed in Hep G2 cells, by transfecting the cells with HBx plasmids, followed by the incubation of HA (50 and 100 μM). In Hep G2 cells, 50 μM HA could not decrease HBx-induced autophagosome formation, beclin-1, c-myc and SIRT-1 expression, which could be due to the difference in the cell line. The results confirmed that HA inhibited the HBx-induced autophagosome formation. Furthermore, HA inhibited HBx-induced c-myc and SIRT-1 expression in Hep G2 cells, confirming that HA inhibits HBV DNA, HBsAg and HBx protein in Hep G2.2.1.5 cells.

A number of compounds isolated from the natural products including, Epigallocatechin-3-gallate has been reported to inhibit the HBV replication via inhibiting the autophagosome formation in HepG2215 cells^26^,^27^,^28^,^29^. Some compounds have been seen to inhibit the viral replication by targeting the HBx protein^30^. A number of the recent investigations suggest several anticipatory approaches, including vaccination, passive immunization, and numerous other helpful treatments for HBV infections are available nowadays. However, other approaches are always required. As HBV particularly modulate the process of autophagy in host cells to enhancing viral replication and virion production^4^. Targeting the autophagy in its different steps that change HBV replication open the new approach for the improvement in novel therapeutic strategy against HBV^26^.

**Figure 8:**
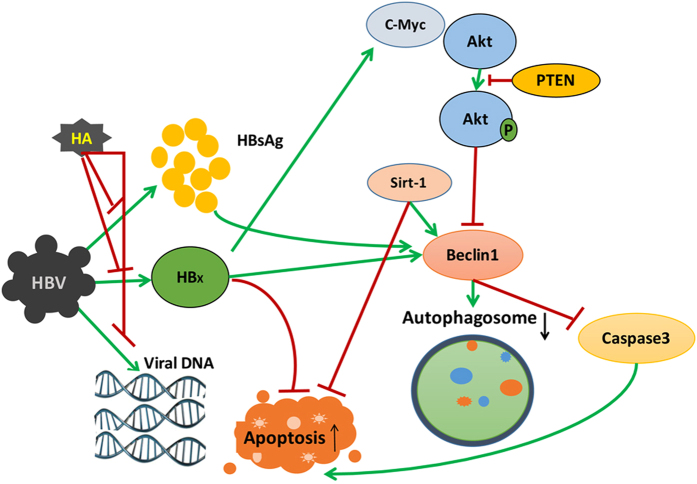
Schematic diagram showing the regulation of HA in HBx-induced proliferation and autophagy. HA inhibits HBx, HBV DNA and HBsAg levels. Further, HA inhibits HBx-induced cell proliferation via c-myc/akt/β-catenin pathway and autophagosome formation via SIRT-1/beclin1 pathway in Hep G2.2.1.5 cells.

In conclusion, the autophagy pathway can be a target for designing drugs that better serve the purpose of patients with HBV infections in the liver. The role of autophagy in HBV infection is summarized in Fig. 8. The data clearly shows that HA inhibits HBx, HBV DNA and HBsAg levels. Further, HA inhibits the HBx-induced cell proliferation via c-myc/akt/β-catenin pathway and autophagosome formation via SIRT-1/beclin1 pathway in Hep G2.2.1.5 cells. Autophagy is closely related to the cell death pathways involving apoptosis and ER stress-mediated cell death. Therefore, targeting autophagy in the infected host cells and inducing cell death could provide another approach to combat against HBV infection. In this study, we provided the first evidence that HA can efficiently inhibit the HBV replication and the expression of HBx protein in HepG2 2.2.15 cells and inhibit viral DNA replication.

## Materials and Methods

### Cell culture and HA treatment

The Hep G2 cells derived Hep G2.2.15 cell lines (Hep G2 cells expressing HBV) were used for all the experiments. Cells were seeded in Dulbecco’s Minimal Essential Media (DMEM) with 5% Foetal Bovine Serum (FBS) and 1 mg/ml penicillin and streptomycin at 37 °C and 5% CO_2_. For HA treatment, 1 × 10^5^ cells of Hep G2 2.2.15 were seeded in 6-well plates and allowed to grow for 24 hours before treatment with different concentrations (50 and 100 μM) of HA.

### Cell Proliferation assay

Cells were inoculated onto a 96-well plate at a density of 5,000 cells per 100 μl 24 hours before the HA treatment. HA was incubated with different concentrations and the cells were further incubated for 24 hours. After the treatment, the proliferation was measured using MTT assay as described previously^14^. The cell viability was calculated as shown in equation 1:


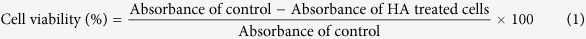


### HBV DNA isolation and quantitation

HBV DNA was isolated from Hep G2 cells and the cell culture supernatant. Briefly, both cell pellet and supernatant (200 μl) was lysed by autoclaved milli Q distilled water (up to 500 μl) followed by addition of TE saturated phenol. The tubes were vortexed thoroughly and incubated at 65 °C for 2 hours. The tubes were centrifuged at 12000 rpm for 10 min and extracted once with 200 μl chloroform. The supernatant was precipitated with 7.5 M ammonium acetate and 100% ethanol. After precipitation, DNA was washed with 70% ethanol, air dried and stored at −20 °C.

HBV DNA quantitation was carried out by real time PCR using Taqman probe (primers HBVx1547F: ACTCCCCGTCTGTGCCTTCT, HBVx1637R: GATCTGGTGGGCGTTCAC and probe HBV1570-5′FAM-CCGGACCGTGTGCACTTCGCTT-BHQ) of X region. Serial dilutions (2–8 log_10_) of HBV DNA High control (Acrometrix, USA) was used as laboratory control for derivation of standard curve. HBV DNA quantity was calibrated using HBV DNA panel (Acrometrix, USA) compared with reference WHO HBV control to express HBV DNA quantity in international units per ml (IU/ml).

### HBsAg ELISA

Hepatitis B surface antigen (HBsAg) in cell culture supernatant was detected by ELISA using Monolisa HBsAg ULTRA Kit (Bio-Rad, USA). Briefly, 100 μl of cell culture supernatant and control (negative/positive) sera was dispensed in wells of microplate. This was followed by addition of 50 μl conjugate to all wells and incubation at 37 °C for 90 minutes. The unbound conjugate was removed by washing 5 times using washing solution. Each well was added with 100 μl substrate/color development solution and incubated for 30 minutes at room temperature. Then 100 μl stopping solution was added to each well and optical density (OD) was read at 450 nm. The cut off value was determined by adding 0.05 to mean OD value of negative controls. The ratio value of sample was calculated by dividing the OD of samples by the cut-off value.

### SDS-PAGE and Western blots

Cells were washed with the PBS. MPER (Thermo) Mammalian protein isolation buffer supplemented with protease inhibitor cocktail was used. The bicinchoninic acid (BCA) protein assay kit using bovine serum albumin (BSA) as the standard determined protein concentration in the cells lysate. SDS-PAGE was used to separate the proteins and transferred to PVDF membrane. These membranes were blocked with 5% non-fat dry milk or BSA in Tris-buffered saline + 0.05% Tween 20 (TBS-T) for 1 h and then incubated with specific primary antibodies in 5% milk or BSA at 4 °C overnight. Three washes with TBS-T was done and the blots were incubated with secondary immunoglobulin G (IgG) for 1 h at room temperature. Chemiluminescence was detected by the help of ECL Western blotting substrate (Thermo), visualized in Polaroid film by X-ray developer.

### Autophagosome formation assay

HepG2 cells were first transfected with HBx plasmid and treated with HA. In parallel experiments, HepG2.2.1.5 cells were also incubated with HA. After 24 h of incubation, autophagosome formation was analysed using autophagosome formation detection kit (Enzo life sciences, USA) as per manufacturer’s instructions. Briefly, diluted microscopy dual detection reagent (100 μl) was added and incubated for 30 minutes followed by DAPI containing mounting solution addition for 15 minutes at room temperature. At least 10 different high-power fields under a fluorescent microscope were counted and the percent of autophagosome formation was calculated using equation 2.


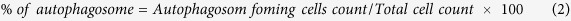


### TUNEL assay

HepG2.2.1.5 cells were plated in 6 well plates (3.5 × 10^5^ cells/well) and challenged with the HA as described above. The cells incubated with 100 mM hydrogen peroxide for 2 hours were used as a positive control. The cells were fixed using 4% paraformaldehyde and then permeabilized solution (0.1% triton-X100 in 0.1% sodium citrate) was added for 15 minutes. Cells were washed with PBS and the TUNEL reagent (Roche, USA) was added and incubated for 1 hour at 37 °C. The mounting solution containing DAPI (Vector labs, USA) was added to the cells. At least 10 different high-power fields under a fluorescent microscope were observed and the data was presented as percent of apoptosis by calculating TUNEL positive cells to a total number of cells as shown in equation 3.


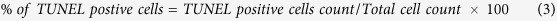


### Statistical analysis

All experiments were performed at least three times (n = 3) in duplicates. The data were expressed as mean ± standard deviation and unpaired Student’s t-test between two groups and one-way ANOVA was performed among groups. p < 0.05 was considered as statistically significant.

## Additional Information

**How to cite this article**: Pant, K. *et al*. Humic acid inhibits HBV-induced autophagosome formation and induces apoptosis in HBV-transfected Hep G2 cells. *Sci. Rep.* **6**, 34496; doi: 10.1038/srep34496 (2016).
